# Racial and Ethnic Variation in Complementary and Integrative Health Therapy Use Among US Veterans

**DOI:** 10.1001/jamanetworkopen.2023.18020

**Published:** 2023-06-16

**Authors:** Jessica Tobin, Steven B. Zeliadt, Dawn M. Upchurch, Rian DeFaccio, Jamie Douglas, Hannah M. Gelman, Matt Hawrilenko, Stephen Frochen, Nathan McGinty, Adam Resnick, Nathan Tomlanovich, Joy Toyama, Alison M. Whitehead, Benjamin Kligler, Stephanie L. Taylor

**Affiliations:** 1 Center for the Study of Healthcare Innovation, Implementation and Policy, Greater Los Angeles VA Healthcare System, Los Angeles, California; 2 Center of Innovation for Veteran-Centered and Value-Driven Care, VA Puget Sound Healthcare System, Seattle, Washington; 3 Department of Health Services, School of Public Health, University of Washington, Seattle; 4 Department of Community Health Sciences, University of California, Los Angeles Fielding School of Public Health, Los Angeles; 5 Office of Patient-Centered Care and Cultural Transformation, Veterans Health Administration, Washington, DC; 6 Department of Medicine, Geffen School of Medicine; University of California, Los Angeles; 7 Department of Health Policy and Management, University of California, Los Angeles Fielding School of Public Health, Los Angeles

## Abstract

**Importance:**

White individuals are the greatest users of complementary and integrative health (CIH) therapies in the general population, but this might partially be due to differences in age, health condition, and location. Identifying the nuances in racial and ethnic differences in care is one important step to addressing them.

**Objective:**

To evaluate racial and ethnic differences in Veterans Affairs (VA)–covered CIH therapy use in a more nuanced manner by examining the association of 5 demographic characteristics, health conditions, and medical facility locations with those differences.

**Design, Setting, and Participants:**

Retrospective cross-sectional observational study of VA health care system users, using electronic health record and administrative data at all VA medical facilities and community-based clinics. Participants included veterans with nonmissing race and ethnicity data using VA-funded health care between October 2018 and September 2019. Data were analyzed from June 2022 to April 2023.

**Main Outcome and Measure:**

Any use of VA-covered acupuncture, chiropractic care, massage therapy, yoga, or meditation/mindfulness.

**Results:**

The sample consisted of 5 260 807 veterans with a mean (SD) age of 62.3 (16.4) years and was 91% male (4 788 267 veterans), 67% non-Hispanic White (3 547 140 veterans), 6% Hispanic (328 396 veterans), and 17% Black (903 699 veterans). Chiropractic care was the most used CIH therapy among non-Hispanic White veterans, Hispanic veterans, and veterans of other races and ethnicities, while acupuncture was the most commonly used therapy among Black veterans. When not accounting for the location of the VA medical facilities in which veterans used health care, Black veterans appeared more likely to use yoga and meditation than non-Hispanic White veterans and far less likely to use chiropractic care, while those of Hispanic or other race and ethnicity appeared more likely to use massage than non-Hispanic White veterans. However, those differences mostly disappeared once controlling for medical facility location, with few exceptions—after adjustment Black veterans were less likely than non-Hispanic White veterans to use yoga and more likely to use chiropractic care.

**Conclusions and Relevance:**

This large-scale, cross-sectional study found racial and ethnic differences in use of 4 of 5 CIH therapies among VA health care system users when not considering their medical facility location. Given those differences mostly disappeared once medical facilities were accounted for, the results demonstrated the importance of considering facilities and residential locations when examining racial differences in CIH therapy use. Medical facilities could be a proxy for the racial and ethnic composition of their patients, CIH therapy availability, regional patient or clinician attitudes, or therapy availability.

## Key Points

**Question:**

Do racial and ethnic differences in the use of complementary and integrative health (CIH) therapies exist, and do those differences vary by gender, age, and chronic conditions among users of the Veterans Affairs (VA) health system?

**Findings:**

In a cross-sectional analysis of more than 5 million veterans, racial and ethnic differences in VA CIH therapy use were found when not considering veterans’ medical facility location. However, those differences mostly disappeared once controlling for VA medical facility location, and the direction of those differences changed.

**Meaning:**

The findings suggest the importance of considering facility, which could be a proxy for the racial and ethnic composition of patients, CIH therapy availability, or regional patient or clinician attitudes, examining racial and ethnic differences in CIH therapy use.

## Teaser

This cross-sectional study evaluates racial and ethnic variation in complementary and integrative health therapy use among US veterans.

## Introduction

Interest in complementary and integrative health (CIH) therapies such as yoga, acupuncture, and meditation has increased as research has demonstrated their effectiveness as fairly low-risk and low-cost approaches to managing chronic health conditions.^1,2,3,4^ As a result, they are being included in national pain management guidelines,^5^ while Congress and Veterans Affairs (VA) leadership have provided strong support for them.^6^ VA is at the forefront of widespread provision of CIH therapies via its Whole Health System Transformation,^7^ which should result in appropriate increased use of CIH therapies.

National studies in the general population find that people in minoritized racial and ethnic groups are less likely to use various CIH therapies than White individuals.^8^ Reasons for these differences may range from lack of access to potential culturally based preferences^9,10^ or affordability.^11^ Given that, unique insights may be generated by exploring CIH therapy use in a setting with somewhat more equitable access, such as the VA, where patients have access to CIH therapies regardless of income. However, regional variation in the VA’s provision of CIH therapies does still exist to a degree.^12^

CIH therapy use also differs by physical and mental health conditions among the general population. Chronic pain, anxiety, and depression are frequent clinical indications for CIH therapy use, with CIH therapies serving as alternatives or supplements to conventional treatments.^5,13,14^ CIH therapies might also be recommended for physical conditions such as cardiovascular disease, obesity, and diabetes or for stress-reduction or low-impact physical activity.^15,16^ These health conditions also often vary in prevalence by race and ethnicity,^17,18^ making them important to consider when exploring racial and ethnic variation in CIH therapy use.

Although racial and ethnic differences in CIH therapy use are documented in national adult samples within and outside the VA, less is known about how these differences may vary by other patient characteristics and conditions or by medical facility location where the therapies are available.^19^ Thus, we examined racial and ethnic variation in the use of 5 of the most frequently used CIH therapies in the VA: yoga, meditation/mindfulness, massage therapy, chiropractic care, and acupuncture. In exploratory analyses of variation in racial and ethnic differences in use by other patient characteristics, we also examined the role of medical facility location and interactions between race and ethnicity and age, gender, chronic pain, anxiety, and depression.

## Methods

Consistent with the Strengthening the Reporting of Observational Studies in Epidemiology (STROBE) reporting guideline for cross-sectional studies, the following describes sample eligibility and study methods.^20^ The VA Greater Los Angeles institutional review board determined this work to be a quality improvement effort for VA operations and thus exempt from review. Participants did not provide informed consent, as the study was restricted to secondary data analysis of routinely collected VA data.

### Participants and Their Medical Facility Location

A nationwide cohort of veterans having a VA visit between October 1, 2018, and September 30, 2019, were included in this cross-sectional analysis if they had a primary care, mental health, or pain clinic visit in the VA electronic health record (EHR) during that time. If a veteran had a qualifying visit at more than 1 VA facility in 2019, they were associated with the facility of the most recent visit.

### Measures

#### CIH Therapy Use

Veterans’ use of CIH therapies was determined using data from VA EHR and from community-based claims. We used 20 codes in 3 data fields (*Current Procedural Terminology*, “Char4”, and “stop” codes) and search terms to capture all CIH therapy utilization data from the VA EMR, the Corporate Data Warehouse. We also used *Current Procedural Terminology *codes from claims data to capture use of CIH therapies that were provided in the community but paid for by the VA, as described in detail elsewhere.^19^ Also, records were excluded if they were associated with no-show visits or did not include the provision of care (eg, referrals, consultations). We examined the 5 most frequently used CIH therapies in the VA, which were acupuncture (either traditional or Battlefield [auricular]^21^), chiropractic care, therapeutic massage, meditation/mindfulness, and yoga. A patient was considered to have used a CIH therapy if they had 1 or more visits for that therapy in the study period.

#### Sociodemographic Characteristics and Health Conditions

Our primary variable of interest was self-reported race and ethnicity (ie, Hispanic or Latino, non-Hispanic White, non-Hispanic Black, non-Hispanic other [other includes Asian, Native Hawaiian or Pacific Islander, American Indian and Alaska Native, and self-identified unknown], and non-Hispanic self-identified unknown), for which data were obtained from the VA EHR. Veterans with missing race and ethnicity were excluded from the analysis. We also examined the association that 2 sociodemographic characteristics and 3 health conditions might have with racial and ethnic disparities in use. Age and gender were chosen given the documented variation in CIH therapy use by these factors, and 3 (ie, chronic pain, depression, and anxiety) health conditions were chosen because they are among the most frequent clinical indications for CIH therapy use.^5,8,12,13^ Chronic pain was determined using an algorithm developed by the VA-Department of Defense Pain Management Collaboratory, which requires 2 criteria be met in the year before the date of their qualifying primary care, mental health, or pain clinic visit.^22^ The first is having 2 moderate-to-severe pain severity scores at least 30 days apart on the numeric rating scale (NRS ≥4) in the year before their qualifying VA utilization visit. The second is a diagnosis of musculoskeletal pain in the EHR, according to a set of *International Statistical Classification of Diseases, Tenth Revision, Clinical Modification (ICD-10-CM)* codes.^23^ Anxiety and depression were determined by documented *ICD-10-CM* diagnoses codes in the year before their qualifying VA utilization visit.^24^

### Statistical Analysis

First, we calculated univariate descriptive statistics for all patient sociodemographic characteristics and health conditions overall and by the CIH therapy used. Then we calculated the differences in these patient characteristics by CIH therapy use using χ^2^ statistics (2-sided hypothesis tests). In multivariable logistic regression models, the association between race and ethnicity and use of CIH therapies was assessed with each therapy modeled separately as the outcome, adjusting for the 5 covariates (age, gender, anxiety, depression, and chronic pain). To explore how medical facility location might be associated with race and ethnic differences in use, we ran models with and without a random intercept to account for site differences (and the nesting of individuals within medical facilities). Also, we excluded from the models people who were missing any data, which were few. Finally, we explored the interactions between each race and ethnicity and the 5 covariates. For increased interpretability, we estimated projected probabilities to generate risk ratios and 95% CIs from model results.^25^ We used the margins suite of commands in Stata version 17 (StataCorp), with covariates set to their observed values, so that estimated effect sizes are adjusted to the distribution of the observed values of the covariates. Data were analyzed from June 2022 to April 2023.

## Results

After excluding 161 468 veterans with missing race and ethnicity, the analytic sample consisted of 5 260 807 veterans and included 4 788 267 male veterans (91%), 3 547 140 non-Hispanic White veterans (67%), 328 396 Hispanic veterans (6%), 903 699 Black veterans (17%), and 481 572 veterans of other race and ethnicity (9%) (Table 1). The mean (SD) age of the cohort was 62.3 (16.4). Differences between those missing and not missing race and ethnicity data are presented in the eTable in Supplement 1. More than 5% of veterans used at least 1 of the 5 therapies examined, with higher rates of use among those with chronic conditions (Table 1). Overall rates of CIH therapy use ranged from 0.3% for each of yoga (14 424 participants), massage therapy (17 983 participants), and meditation/mindfulness (15 316 participants) to 2.6% for acupuncture (134 941 participants) and 3.0% for chiropractic care (159 505 participants). Chiropractic care was the most commonly used among White veterans (107 919 participants [3.0%]), Hispanic veterans (12 230 participants [3.7%]), and veterans of other race and ethnicity (16 079 participants [3.3%]), while acupuncture was the most commonly used therapy among Black veterans (25 394 participants [2.8%]). In adjusted main effects models that did not account for medical facility location (Table 2), we found that relative to White veterans, Black veterans were more likely to use yoga and meditation and less likely to use all other CIH therapies, Hispanic veterans were more likely to use massage therapy and less likely to use acupuncture and chiropractic care, and veterans of other race and ethnicity were more likely to use massage therapy and chiropractic care but less likely to use acupuncture. However, once we included the random effects term to control for the nesting of veterans within their medical facilities and to explore the role of medical facility on race and ethnic differences in use, we found almost no differences. Yoga was the only CIH therapy that still had differences in use. Specifically, Black or Hispanic veterans were less likely than non-Hispanic White veterans to use yoga. However, those differences were negligible (although significant, due to the very large sample size).

**Table 1.** Patient Characteristics by Complementary and Integrative Health Therapy Use

Characteristic	Analytic sample, No. (column %) (N = 5 260 807)^a^	No. (row %)				
		Any yoga use (n = 14 424 [0.3])	Any meditation/mindfulness use (n = 15 316 [0.3])	Any therapeutic massage use (n = 17 983 [0.3])	Any acupuncture use (n = 134 941 [2.6])	Any chiropractic care use (n = 159 505 [3.0])
Gender						
Male	4 788 267 (91.0)	10 607 (0.2)	12 182 (.3)	15 144 (.3)	109 946 (2.3)	132 787 (2.8)
Female	472 540 (9.0)	3817 (0.8)	3134 (.7)	2839 (.6)	24 995 (5.3)	26 718 (5.7)
Race and ethnicity						
Non-Hispanic White	3 547 140 (67.4)	8330 (0.2)	9143 (0.3)	10 386 (0.3)	86 179 (2.4)	107 919 (3.0)
Hispanic	328 396 (6.2)	1038 (0.3)	1172 (0.4)	2911 (0.9)	10 345 (3.2)	12 230 (3.7)
Non-Hispanic Black	903 699 (17.2)	3946 (0.4)	3915 (0.4)	3105 (0.3)	25 394 (2.8)	23 277 (2.6)
Non-Hispanic other^b^	481 572 (9.2)	1110 (0.2)	1086 (0.2)	1581 (0.3)	13 023 (2.7)	16 079 (3.3)
Age category, y						
18-39	693 441 (13.2)	2430 (0.4)	2491 (0.4)	2961 (0.4)	21 817 (3.2)	41 694 (6.0)
40-49	501 314 (9.5)	2186 (0.4)	2191 (0.4)	2537 (0.5)	21 076 (4.2)	29 223 (5.8)
50-59	757 908 (14.4)	3290 (0.4)	3538 (0.5)	3608 (0.5)	28 793 (3.8)	31 532 (4.2)
60-69	1 189 311 (22.6)	3597 (0.3)	3937 (0.3)	4174 (0.4)	31 280 (2.6)	30 114 (2.5)
≥70	2 188 833 (40.3)	2921 (0.1)	3159 (0.2)	4703 (0.2)	31 975 (1.5)	26 942 (1.3)
Anxiety	692 075 (13.2)	4614 (0.7)	5535 (0.8)	3863 (0.6)	32 385 (4.7)	35 071 (5.1)
Depression	902 415 (17.2)	6800 (0.8)	7549 (0.8)	5274 (0.6)	44 693 (5.0)	43 489 (4.8)
Chronic pain	1 195 711 (22.7)	7901 (0.7)	7983 (0.7)	10 957 (0.9)	86 608 (7.2)	81 140 (6.8)

^a^ Overall, 10 199 (0.002%) were missing data on health conditions, 203 (0.00004%) were missing age, and 161 468 (3%) missing race and ethnicity. Rates of use of each CIH therapy differed significantly by all patient characteristics at *P* < .001.

^b^ Other includes Asian, Native Hawaiian or Pacific Islander, American Indian and Alaska Native, and self-identified unknown.

**Table 2.** Adjusted Risk Ratios of Complementary and Integrative Health Therapy Use by Racial and Ethnic Group, Relative to Non-Hispanic Whites

Race and ethnicity	Risk ratio vs non-Hispanic White (95% CI)^a^				
	Acupuncture^b^	Chiropractic care^b^	Massage^b^	Meditation^b^	Yoga^b^
Without random effects controlling for medical facility location					
Hispanic	0.87 (0.84-0.90)	0.67 (0.62-0.71)	2.01 (1.94-2.08)	1.00 (0.90-1.10)	0.99 (0.92-1.07)
Black	0.86 (0.84-0.88)	0.58 (0.57-0.59)	0.65 (0.63-0.67)	1.13 (1.07-1.20)	1.22 (1.17-1.27)
Other	0.88 (0.95-0.90)	1.31 (1.28-1.35)	1.75 (1.66-1.84)	0.90 (0.76-1.04)	1.05 (0.94-1.16)
With random effects controlling for medical facility location					
Hispanic	0.99 (0.98-1.00)	1.00 (0.99-1.01)	0.98 (0.96-0.99)	0.98 (0.97-1.00)	0.96 (0.95-0.97)
Black	1.03 (1.03-1.04)	1.07 (1.07-1.08)	1.00 (0.99-1.01)	0.94 (0.94-0.95)	0.93 (0.92-0.94)
Other	1.00 (0.99-1.01)	1.01 (1.00-1.02)	1.00 (0.98-1.01)	1.01 (0.99-1.02)	1.00 (0.99-1.01)

^a^ Rates of use of each CIH therapy differed significantly by race and ethnicity at *P* < .0000.

^b^ Models included the 5 covariates (age, gender, anxiety, depression, and chronic pain).

## Discussion

The VA health care system offers a unique opportunity to examine utilization of CIH therapies given it provides those therapies as care to veterans. As such, it provides a setting with somewhat more equitable access, given patients may obtain services regardless of income. Using our large national data set^19^ of veteran use of VA-covered CIH therapy, we found chiropractic care was the most commonly used CIH therapy among non-Hispanic White veterans, Hispanic veterans, and veterans of other races and ethnicities, while acupuncture was the most commonly used therapy among Black veterans. The overall rates of CIH therapy utilization in VA we found are lower than national estimates, but that could be due to our capturing only VA-provided CIH therapies and the demographic makeup of VA patients; the greatest users of CIH therapies in the general population are younger White women, whereas the VA population is predominantly older and mostly male.^8^

In facility-unadjusted analyses it appeared that Black veterans were more likely to use yoga and meditation than non-Hispanic White veterans, while those of Hispanic or other race and ethnicity were more likely to use massage than non-Hispanic White veterans. However, almost all race and ethnic differences we first observed mostly disappeared once controlling for medical facility location, with the exception of yoga. Some of these differences might be explained by racial and ethnic differences in VA facilities’ patient population, CIH therapy availability, or cultural or other individual-level differences in treatment preferences among patients or their clinicians.^26,27,28^ If Black veterans were more likely to use yoga in facility-unadjusted analyses but the difference was attenuated when adjusting for site, this suggests that yoga may be provided and/or used more overall at sites with higher proportions of Black veterans. Likewise, Black veterans were much less likely to use chiropractic care in unadjusted models but slightly more likely to use it in the facility-adjusted model, implying that chiropractic care is likely more widely available at sites with lower proportions of Black veterans.

Although increased availability of CIH therapies could benefit a wide range of patients, lack of widespread institutional support for these therapies may be a potential barrier in other settings. The VA has been undergoing a system-wide transformation focused on holistic, person-centered care for all veterans, with support at the highest levels of leadership. This represents a cultural shift which may contribute to increased CIH therapy uptake by positioning CIH therapies within the system as a commonly used, widely available, and broadly supported strategy. Although patient treatment preferences are highly personal, it may be beneficial to promote awareness of CIH therapies for all patients, both those in need of chronic disease management tools as well as generally healthy patients interested in enhancing disease prevention and promoting well-being. In settings where CIH therapies are still viewed and treated as an add-on service inconsistently covered by insurance, patients may not be as aware of or able to afford participating in these therapies. Systems-based efforts to increase access to CIH therapies in other settings may help improve patient knowledge, interest, and uptake beyond the VA.

### Limitations

This study was limited by several factors. Although we adjusted for covariates with ample prior evidence of association with either race and ethnicity and/or CIH therapy use, other unmeasured confounders may have affected our findings, such as socioeconomic status, a variable that is not directly measured at the individual level in VA data. This study examined VA-funded care, so use of CIH therapies in other settings, where patterns of use by patient characteristics may differ than in VA settings, was not captured. In addition, this study’s cross-sectional nature precludes causal inference.

## Conclusions

In this cross-sectional study of veterans’ use of VA-covered acupuncture, chiropractic care, therapeutic massage, meditation/mindfulness, and yoga, we found almost no evidence of racial and ethnic differences in use of the first 4 therapies once we adjusted for the VA medical facility in which veterans received their health care. This points to the importance of considering facilities and residential locations when examining racial differences in CIH therapy use. Facility location could be a proxy for facility racial and ethnic composition, regional patient or clinician attitudes, or therapy availability, so a direct examination of these issues is warranted.

## Notes

Supplement 1. **eTable. **Differences in Observable Characteristics by Missingness on Race and Ethnicity

Supplement 2.
Data Sharing Statement

## References

[1] Chen KW, Berger CC, Manheimer E, . Meditative therapies for reducing anxiety: a systematic review and meta-analysis of randomized controlled trials. Depress Anxiety. 2012;29(7):545-562. doi:10.1002/da.21964 22700446 PMC3718554

[2] Cramer H, Lauche R, Langhorst J, Dobos G. Yoga for depression: a systematic review and meta-analysis. Depress Anxiety. 2013;30(11):1068-1083. doi:10.1002/da.22166 23922209

[3] Kukimoto Y, Ooe N, Ideguchi N. The effects of massage therapy on pain and anxiety after surgery: a systematic review and meta-analysis. Pain Manag Nurs. 2017;18(6):378-390. doi:10.1016/j.pmn.2017.09.001 29173797

[4] Manyanga T, Froese M, Zarychanski R, . Pain management with acupuncture in osteoarthritis: a systematic review and meta-analysis. BMC Complement Altern Med. 2014;14(1):312. doi:10.1186/1472-6882-14-312 25151529 PMC4158087

[5] Qaseem A, Wilt TJ, McLean RM, ; Clinical Guidelines Committee of the American College of Physicians. Physicians* CGCotACo. Noninvasive treatments for acute, subacute, and chronic low back pain: a clinical practice guideline from the American College of Physicians. Ann Intern Med. 2017;166(7):514-530. doi:10.7326/M16-2367 28192789

[6] Expanding the VA Whole Health System: Whole Health. Accessed June 3, 2022. https://www.va.gov/WHOLEHEALTH/features/Expanding_the_VA_Whole_Health_System.asp

[7] Kligler B, Hyde J, Gantt C, Bokhour B. The Whole Health Transformation at the Veterans Health Administration: moving from “What’s the matter with you?” to “What matters to you?”. Med Care. 2022;60(5):387-391. doi:10.1097/MLR.0000000000001706 35283434

[8] Clarke TC, Barnes PM, Black LI, Stussman BJ, Nahin RL. Use of yoga, meditation, and chiropractors among US adults aged 18 and over. NCHS Data Brief. 2018;(325):1-9. 30475686

[9] Elewonibi BR, BeLue R. Prevalence of complementary and alternative medicine in immigrants. J Immigr Minor Health. 2016;18(3):600-607. doi:10.1007/s10903-015-0210-4 25921731

[10] Zörgő S, Purebl G, Zana Á. A qualitative study of culturally embedded factors in complementary and alternative medicine use. BMC Complement Altern Med. 2018;18(1):25. doi:10.1186/s12906-018-2093-0 29357855 PMC5778786

[11] Farmer MM, McGowan M, Yuan A, Whitehead A, Osawe U, Taylor SL. The organization of complementary and integrative health practices at the VA: a national survey. J Altern Complement Med. 2021;27(S1):124-130. doi:10.1089/acm.2020.0395 33788607

[12] Saper R. Integrative medicine and health disparities. Global Advances in Health and Medicine. 2016;5(1):5-8. doi:10.7453/gahmj.2015.133 26937308 PMC4756775

[13] van der Watt G, Laugharne J, Janca A. Complementary and alternative medicine in the treatment of anxiety and depression. Curr Opin Psychiatry. 2008;21(1):37-42. doi:10.1097/YCO.0b013e3282f2d814 18281839

[14] Deligiannidis KM, Freeman MP. Complementary and alternative medicine therapies for perinatal depression. Best Pract Res Clin Obstet Gynaecol. 2014;28(1):85-95. doi:10.1016/j.bpobgyn.2013.08.007 24041861 PMC3992885

[15] Birdee GS, Yeh G. Complementary and alternative medicine therapies for diabetes: a clinical review. Clin Diabetes. 2010;28(4):147-155. doi:10.2337/diaclin.28.4.147

[16] Okonta NR. Does yoga therapy reduce blood pressure in patients with hypertension? An integrative review. Holist Nurs Pract. 2012;26(3):137-141. doi:10.1097/HNP.0b013e31824ef647 22517349

[17] Coleman KJ, Stewart C, Waitzfelder BE, . Racial-ethnic differences in psychiatric diagnoses and treatment across 11 health care systems in the mental health research network. Psychiatr Serv. 2016;67(7):749-757. doi:10.1176/appi.ps.201500217 27079987 PMC4930394

[18] Campbell CM, Edwards RR. Ethnic differences in pain and pain management. Pain Manag. 2012;2(3):219-230. doi:10.2217/pmt.12.7 23687518 PMC3654683

[19] Taylor SLGH, DeFaccio R, Hawrilenko M, . Compendium on use of complementary and integrative health therapies and chiropractic care at the VA. Volume 1: use and characteristics of users, fiscal years 2017-2019. Department of Veterans Affairs, Office of Patient Centered Care and Cultural Transformation. 2020. Accessed May 9, 2023. https://www.va.gov/WHOLEHEALTH/docs/CIH_Compendium_Volume1_2020_APR122021_508C.pdf

[20] von Elm E, Altman DG, Egger M, Pocock SJ, Gøtzsche PC, Vandenbroucke JP; STROBE Initiative. The Strengthening the Reporting of Observational Studies in Epidemiology (STROBE) statement: guidelines for reporting observational studies. Ann Intern Med. 2007;147(8):573-577. doi:10.7326/0003-4819-147-8-200710160-00010 17938396

[21] Taylor SL, Giannitrapani KF, Ackland PE, . The implementation and effectiveness of battlefield auricular acupuncture for pain. Pain Med. 2021;22(8):1721-1726. doi:10.1093/pm/pnaa474 33769534

[22] Kerns RD, Brandt CA, Peduzzi P. NIH-DoD-VA pain management collaboratory. Pain Med. 2019;20(12):2336-2345. doi:10.1093/pm/pnz186 31807788 PMC6895460

[23] Goulet JL, Kerns RD, Bair M, . The musculoskeletal diagnosis cohort: examining pain and pain care among veterans. Pain. 2016;157(8):1696-1703. doi:10.1097/j.pain.0000000000000567 27023420 PMC4949131

[24] Quan H, Sundararajan V, Halfon P, . Coding algorithms for defining comorbidities in ICD-9-CM and ICD-10 administrative data. Med Care. 2005;43(11):1130-1139. doi:10.1097/01.mlr.0000182534.19832.83 16224307

[25] Kleinman LC, Norton EC. What’s the Risk? A simple approach for estimating adjusted risk measures from nonlinear models including logistic regression. Health Serv Res. 2009;44(1):288-302. doi:10.1111/j.1475-6773.2008.00900.x 18793213 PMC2669627

[26] Noël LT. An ethnic/racial comparison of causal beliefs and treatment preferences for the symptoms of depression among patients with diabetes. Diabetes Educ. 2010;36(5):816-827. doi:10.1177/0145721710380145 20876308

[27] Pillay T, van Zyl HA, Blackbeard D. Chronic pain perception and cultural experience. Procedia Soc Behav Sci. 2014;113:151-160. doi:10.1016/j.sbspro.2014.01.022

[28] Narayan MC. Culture’s effects on pain assessment and management. Am J Nurs. 2010;110(4):38-47. doi:10.1097/01.NAJ.0000370157.33223.6d 20335689

