# Supplementary material for: Racial and Ethnic Variation in Complementary and Integrative Health Therapy Use Among US Veterans
Source: JAMA Netw Open. 2023 Jun 16;6(6):e2318020. doi: 10.1001/jamanetworkopen.2023.18020 (PMC10276309; doi:10.1001/jamanetworkopen.2023.18020)
Supplement: Supplement 1. — eTable. Differences in Observable Characteristics by Missingness on Race and Ethnicity [file jamanetwopen-e2318020-s001.pdf]

Supplemental Online Content

Tobin J, Zeliadt SB, Upchurch DM, et al. Racial and ethnic variation in complementary and integrative health therapy use among US veterans. *JAMA Netw Open*. 2023;6(6):e2318020. doi:10.1001/jamanetworkopen.2023.18020

eTable. Differences in Observable Characteristics by Missingness on Race and Ethnicity

This supplemental material has been provided by the authors to give readers additional information about their work.

© 2023 Tobin J et al. *JAMA Network Open.*

eTable. Differences in Observable Characteristics by Missingness on Race and Ethnicity

	Non-missing race/ethnicity N=5,260,807	Missing race/ethnicity N=161,468
Characteristic/therapy use	N (%)	
Gender		
Female	472,540 (9)	15,339 (10)
Male	4,788,267 (91)	146,129 (90)
Age category		
18-39	683,441 (13)	25,835 (16)
40-49	501,314 (10)	16,147 (10)
50-59	757,908 (14)	20,991 (13)
60-69	1,189,311 (23)	32,294 (20)
70+	2,188,833 (40)	66,201 (41)
Age unknown	0 (0)	2 (0)
Cardiovascular diseases	3,162,654 (60)	88,807 (55)
Anxiety	692,075 (13)	519,376 (12)
Depression	902,415 (17)	24,220 (15)
PTSD	944,799 (18)	27,449 (17)
Chronic pain	1,195,711 (23)	30,678 (19)
Obesity	917,498 (17)	25,834 (16)
Diabetes	1,275,918 (24)	35,523 (22)
Chiropractic use	159,505 (3.0)	4,683 (2.9)
Acupuncture use	134,941 (2.6)	3,875 (2.4)
Massage therapy use	17,983 (0.3)	484 (0.3)
Meditation use	15,316 (0.3)	323 (0.2)
Yoga use	14,424 (0.3)	319 (0.2)

© 2023 Tobin J et al. *JAMA Network Open.*
