# Supplementary material for: Racial and Ethnic Variation in Complementary and Integrative Health Therapy Use Among US Veterans
Source: JAMA Netw Open. 2023 Jun 16;6(6):e2318020. doi: 10.1001/jamanetworkopen.2023.18020 (PMC10276309; doi:10.1001/jamanetworkopen.2023.18020)
Supplement: Supplement 2. — Data Sharing Statement [file jamanetwopen-e2318020-s002.pdf]

Data Sharing Statement

Tobin. Racial and Ethnic Variation in Complementary and Integrative Health Therapy Use Among US Veterans. *JAMA Netw Open*. Published June 16, 2023.
doi:10.1001/jamanetworkopen.2023.18020

Data
Data available: No

Additional Information
Explanation for why data not available: VA data are not publicly available.
